# The effects of general warm up, specific warm up and taping on electrical activity of lower limb’s muscles in reaction to sudden unloading while walking

**DOI:** 10.1186/1757-1146-7-S1-A130

**Published:** 2014-04-08

**Authors:** Tolue Sahari, Nader Farahpour, Hamidreza Mokhtarinia, Leyla bavafa

**Affiliations:** 1 Islamic Azad University, Broojerd branch, Broojerd Iran; 2 Bu Ali Sina University, Hamedan, Iran; 3 Islamic Azad University, Hamedan branch, Hamedan, Iran; 4 Rehabilitation University of Tehran, Iran

## Background

Ability of balance maintenance and postural control is important to prevent falling and injuries when balance perturbations occurs during walking. Injuries due to unloading of the base of support in walking and running such as ankle sprain has high incidence. Poor muscle function may put the joint in unstable condition. The association of the unloading and poor muscle function in the ankle joint may increase the rate and the intensity of the ankle sprain. Various techniques have been applied to maximize the joint’s stability. The objectives of this study was to compare the effects of general warming, local warming and taping of the ankle joint on the electrical activity of few muscles during a sudden unloading of the base of support during walking.

## Method

Ten healthy volunteer women (age: 25.6±2.7_Yrs_; height: 163.3±5.6_cm_; mass: 60.3±7.0_kg_) were selected from local population. Using Biometrics Datalog EMG system with eight bipolar surface electrodes the electrical activity of medial gastrcnemious (MG), proneus longus (PL), vastus medialis (VM) and erector spina at L3 l3v3l (ES_L3_) muscles were recorded by 1500_HZ_ sampling frequency. Then signals were filtered using a bound pass filter of 10-500 _HZ_ as well as a filter of 50_HZ_ to eliminate the noise from the city electricity. A 12 meter walkway was made of 24 wooden plates (50_cm_ × 50_cm_ × 4_cm_). Subjects walked through the walkway during the experimentation while each time one of the plates was randomly removed to impose unloading to the leg. Plates were covered with a uniform carpet.Subject was unaware of when and where the unloading will happen. Four different conditions were performed including a) walking without warming up, b) walking after a general warming, c) walking after local warming and d) walking with taping of the ankle. Each condition was repeated five times and its average was used for statistical calculations. The average RMS of every muscle was normalized based on the related maximum RMS obtained by MVIC test. Repeated measure analysis of variance (α<0.05) was used for statistical calculations.

## Results and discussion

During walking, there was not any significant differences on the activity of different muscles (p>0.05). In general unloading resulted in higher EMG amplitude of muscles. There was a significant interaction between unloading and muscle factors (p= 0.001). All muscles except ES_L3_ were influenced by unloading. Warming did not have any significant effects on EMG amplitudes of muscles in both normal and unloading conditions (p>0.05). Symmetrical muscle activity was observed in all conditions by means of similar activity in both the right and the left side’s muscles. This study indicates that warming and taping could not reduce the perturbation effects on muscle activity. This could have two implications. First, the warming intensity was not enough and/or the time between warming and testing was relatively long so that the warming effects were disappeared prior to the test. Taping the ankle did not influence the leg and thigh muscles’ activity. It seems that the perturbation was compensated at lower limb’s muscle and therefore, loading did not have influence on ES_L3_ activity.

## Conclusion

Unloading increases the lower limb’s muscle activity. Lumbar erector spinae muscle was not influenced by unloading. Muscle reaction was not benefited from the proposed warming exercises and taping. It seems that lower limbs’ muscles could compensate the imposed unloading effects. To prevent individuals from ankle sprain due to unloading, it is suggested to examine the interaction between shoe and different warming programs in larger sample size.

